# Differential immunological effects of silica nanoparticles on peripheral blood mononuclear cells of silicosis patients and controls

**DOI:** 10.3389/fimmu.2022.1025028

**Published:** 2022-10-13

**Authors:** Nirosha Ganesan, Steven Ronsmans, Peter Hoet

**Affiliations:** ^1^ Laboratory of Toxicology, Unit of Environment & Health, Department of Public Health and Primary Care, KU Leuven, Leuven, Belgium; ^2^ Laboratory of Respiratory Diseases and Thoracic Surgery (BREATHE), KU Leuven, Leuven, Belgium; ^3^ Clinic for Occupational and Environmental Medicine, Department of Respiratory Diseases, University Hospitals Leuven, Leuven, Belgium

**Keywords:** silicosis, silica nanoparticles, lymphocyte proliferation, CFSE, B cells, PBMC, granuloma, *ex vivo*

## Abstract

Silicosis is a fibrotic disease caused by the inhalation of respirable silica particles, which are typically engulfed by alveolar macrophages and subsequently induce the release of inflammatory cytokines. Various animal experimental and human studies have focused on modeling silicosis, to assess the interactions of macrophages and other cell types with silica particles. There is still, however, limited knowledge on the differential response upon silica-exposure between silicosis patients and controls. We focused on studying the responsiveness of peripheral blood mononuclear cells (PBMCs) to silica nanoparticles (SiNPs) - Ludox and NM-200 - of silicosis patients and controls. The proliferative capacity of T- CD3^+^ and B- CD19^+^ cells, were evaluated *via* Carboxyfluorescein succinimidyl ester (CFSE) assay. The activation status of lymphocyte subsets and response to silica were also evaluated by comparing the extent of micro-granuloma or aggregate formation with the cytokine secretion profiles between both groups of individuals. The proliferative capacity of CD19^+^ cells was elevated in silicotic patients as opposed to controls. Subsets of regulatory T cells (CD4^+^ CD25^+^ and CD8^+^ CD25^+^) and immunoglobulins IgM and IgG were also significantly increased in patients. The number and the size of aggregates formed were higher with SiNPs stimulation in patients compared to controls. Multivariable analysis also elucidated the role of key cytokines like interleukin-1β (IL-1β), IL-6 and interferon-gamma (IFN-γ), which were upregulated in SiNP-stimulated PBMCs of patients compared to controls. Our *ex vivo* model thus has potential to provide insights into the immunological effects of silica particles in lymphocytes of silicosis patients and controls.

## Introduction

Silicosis is a fibrotic lung disease caused by the inhalation of respirable crystalline (<10µm; SiO_2_) (1) and some amorphous forms of silica (2). Exposure to respirable crystalline silica can occur in a range of occupational settings such as in mines, quarries, iron foundries, and construction industry, especially in jobs that involve cutting, sawing, grinding, drilling, and crushing of natural or silica-based artificial stones. Diagnosis of silicosis generally relies on a history of substantial exposure to silica dusts and compatible radiological features, together with the exclusion of other diagnoses (1). The typical histologic findings include hyaline nodules (silicotic nodules) in lung and hilar/mediastinal lymph nodes (1). Loss of lung function has been associated with magnitude of exposure and extent of radiological lesions (3). Currently, there is no effective treatment to silicosis and lung transplantation remains the only option for patients with terminal silicosis (4). Silicosis is still prevalent in developing countries with low socio-economic status where the cases are still under-reported due to poor surveillance (1). At the same time, silicosis still remains a major occupational health concern, in developed continents like Europe and the Americas, despite the prevention efforts by many countries worldwide (1). In addition, silica exposure has been associated with the development of tuberculosis, chronic obstructive pulmonary disease (COPD) and autoimmune disorders such as systemic sclerosis and rheumatoid arthritis (1).

Inhaled silica particles that reach the alveoli are typically engulfed by macrophages (5), resulting in macrophage activation and apoptosis (6). Subsequently, the release of inflammatory cytokines and other epithelium damaging substances induce proliferation of fibroblasts, which can ultimately lead to silicosis (5). Various animal and *in vitro* experiments have thus focused on modeling silicosis to assess the interactions of macrophages and other cell types with silica particles (1). In animal studies, both T helper type 1 (Th1) and 2 (Th2) polarization have been found to be associated with silicosis development (7). M1 macrophages have been found to be dominant in the early inflammation stage and accompanied by high expression of the inflammatory cytokines tumor necrosis factor-alpha (TNF-α), interleukin-1β (IL-1β), and IL-6, while M2 macrophages appear dominant in the late fibrosis stage, accompanied by high expression of the anti-inflammatory cytokine IL-10 (8). An increased percentage of activated T cells has also been described in a silicosis rat model (9) and in patients with silicosis (10). Additionally, increased levels of soluble interleukin-2 receptor (sIL-2R), a heterotrimeric protein that is upregulated on activated T cells; have been observed in silica-exposed individuals (11).

When lymphocytes from healthy individuals were challenged with silica nanoparticles <10nm, significantly increased expression of activation markers: CD69^+^ on CD4^+^ and CD8^+,^ and CD25^+^ on CD8^+^ were noted (12). At the same time, silica nanoparticles <10nm induced minimal proliferation of CD4^+^ and CD8^+^ (12). Isolated lymphocytes from silica-exposed individuals showed a significant increase in spontaneous (without antigen or silica stimulation) production of pro-inflammatory Th1 cytokines like IL-1β, IL-6, and TNF-α (11) compared with controls. At the same time, Th2 cytokines IL-10 and transforming growth factor beta (TGF-β), were increased in cell cultures of these silica-exposed workers (11). However, it remains unknown if lymphocytes obtained from silicosis patients will in fact react differently from controls upon stimulation with silica *ex vivo*/*in vitro*.

In this study, we studied the *ex vivo* immune effects of silica on peripheral blood mononuclear cells (PBMCs) from silicosis patients and healthy individuals. Two types of silica nanoparticles (SiNPs): colloidal silica, Ludox, and synthetic amorphous silica, NM-200, were selected as stimuli.

After SiNP exposure, cellular staining of PBMCs and characterization of lymphocyte subsets *via* surface markers were performed, to evaluate extent of proliferation by assessing overall and subset-specific stimulation index (SI) and activation with CD25^+^, respectively. To investigate the modulation by SiNPs and cellular morphology of granulomatous structures, we applied an *ex vivo* model of human silicotic micro-granulomas as described by Ganesan et al. (13). Additionally, the cytokine release profiles and immunoglobulin levels across the different exposures to SiNPs were investigated.

## Materials and methods

### Human blood samples

Whole blood (40-50 ml) was obtained from eight male (four silicotic and four healthy) subjects upon informed consent. Subjects were recruited *via* the outpatient Clinic for Occupational and Environmental Medicine of the University Hospitals Leuven (Belgium). The study was approved by the Ethics Committee Research UZ/KU Leuven (S61777).

### Isolation of human PBMCs

Human PBMCs were isolated by Lymphoprep™ (STEMCELL) density gradient centrifugation. Whole blood was first diluted 1:2 with phosphate-buffered saline (PBS) and poured into a 12 ml Leucosep™ tube (Greiner Bio-One) containing 3 ml of Lymphoprep™ at a ratio of 1.5:1 and centrifuged at 20°C for 15 min at 400 g, with centrifuge brakes off. PBMCs were removed carefully and washed three times with PBS for 10 min at 400 g, 20°C. Viability and cell counting of freshly acquired PBMCs was performed with trypan blue exclusion assay. A final PBMC concentration of 1 X 10^6^ cells/ml was then prepared by resuspending cells in serum-free PBS.

### Study outcomes

Lymphocyte proliferation and activation, immunoglobulin (IgM and total IgG) levels, number and size of granulomatous structures, cytokine secretion levels and cellular viability.

### Treatment

#### Synthetic amorphous silicon dioxide (Silica nanoparticles; SiNPs)

SM30 Ludox^®^ colloidal silica (L10) was purchased from Sigma-Aldrich as 30 wt % suspension in H_2_O. A starting stock solution of 1000 µg/ml Ludox in 0.05% bovine serum albumin (BSA) in distilled water (dH_2_O) was prepared.

The European Commission’s Joint Research Centre (JRC, Italy) kindly provided NM silica JRCNM02000 (NM-200). Ultrasonic probe sonication was performed with 0.0153g of NM-200 dispersed in 30 µl of ethanol and 5.7 ml 0.05% BSA in dH_2_O for 15 min 20 sec. Physical properties of both Ludox and NM-200 are detailed in **Table 1**. Additional detailed physico-chemical characterization in their manufactured form is provided in the technical report of JRC (14) and by Sigma-Aldrich (15).

**Table 1:** Physical properties of amorphous silica nanoparticles.

SiO_2_	Ludox (L10)	NM-200
Primary particle size (nm)	9 ± 3	15 - 20
Equivalent spherical diameter (nm)	7	22
(DLS) Z-average size (nm)	14 ± 4	175.9 ± 4.5
Polydispersity (PDI)	(P10: 8.1 - P90: 11.8)	0.355
Specific surface area (m^2^/g)	345	204.11
Zeta Potential (mV)	-26.3	-43.7

#### Experimental setup

A total of about 1.8 X 10^5^ PBMCs/well (96-well U-bottom culture plates) and 6 X 10^5^ PBMCs/well (96-well cell flat-bottom culture plates) were suspended in Roswell Park Memorial Institute (RPMI) 1640 medium supplemented with 10% autologous human serum (10% HS) before culture in RPMI 1640 medium supplemented with 10% HS, 1% penicillin-streptomycin (P-S) (100 U/mL), 1% L-glutamine (2 mM) and 1% fungizone (2.5 g/mL) at 37°C in a 5% CO_2_ environment. PBMC samples were stimulated for 7 days by five different concentrations of SiNPs (0.01, 0.1, 1, 10 and 100 µg/ml). A positive control, pokeweed mitogen (PWM, data not shown) and negative control (unstimulated condition) were additionally tested across all samples. Samples in U-bottomed wells were used to detect proliferation and activation of lymphocytes, immunoglobulin levels and cytokine secretion levels. All exposure conditions were performed as triplicates. In case PBMC yield of a sample was insufficient, experiments were still performed in triplicates but with the omission of lower concentrations of SiNPs (0.01 and 0.1 µg/ml).

Samples in flat-bottomed wells were used to detect aggregate formation and a minimum of two replicates per exposure condition was included. During the exposure period, aggregate formation was examined under an Olympus light microscope and imaged with an EVOS cell imaging system (ThermoFisher Scientific) at 10x magnification (TRANS), at 24-hour intervals. On days 3, 5 and 7, cytospin slides of cells stimulated with SiNPs were prepared and stained with hematoxylin and eosin (H&E) stain.

#### Measurement of lymphocyte proliferation *via* CFSE assay

Fluorescein isothiocyanate (FITC)-Carboxyfluorescein succinimidyl ester (CFSE; CellTrace™ CFSE proliferation kit, ref. C34554, ThermoFisher Scientific) labelling of PBMCs was performed at a 1:1 ratio with a final concentration of CFSE at 2.5 µg/ml and allowed to incubate at room temperature (RT) for 8 min while protected from light. CFSE labelled PBMCs were then washed twice before incubation.

#### CFSE assay flow cytometric analysis

Following the incubation period, experiments were halted on the 7^th^ day and the cell suspension was stained with FVD-780 for 30 min at RT. Cell suspension was then resuspended in an antibody cocktail with combinations of Alexa Fluor 700 (AF700) anti-CD3 (UCHT1), allophycocyanin (APC) anti-CD4 (RPA-T4), eFluor 450 (e450) anti-CD8 (RPA-T8), peridinin chlorophyll Cy5.5 (PerCP-Cy5.5) anti-CD19 (HIB19), and phycoerythrin (PE) anti-CD25 (M-A251). This suspension was incubated for 25 min at 4°C while protected from light. Following the washing step, cell suspension was then resuspended in 1% paraformaldehyde (PFA) and incubated for 20 min at 4°C while protected from light. After 20 min, cell suspension was washed in PBS and resuspended in 100 µl of 0.05% BSA in PBS and processed within 1-3 days.

The median fluorescence intensity (MFI) levels of CFSE labelled PBMCs were measured using BD LSR Fortessa and twenty thousand events were collected. Data acquired was analyzed using FlowJo software. Compensation matrix was always performed for each experiment on FlowJo, prior to gating strategy (see **Supplementary Methodology** and **Figure S4**).

Lymphocyte proliferation results were expressed as Stimulation Index (SI)—i.e., the percentage of cells that have proliferated in a stimulated condition divided by the percentage of proliferated cells in the unstimulated condition, on Day 7. An SI ≥ 2.5 was considered as a positive response, in line with several studies concerning beryllium [SI >2.5 (16), and SI >3 (17),], chromium [SI >2 (18, 19),], cobalt [SI >2 (18, 19),] or nickel [SI > 2 (18, 19), and SI >5.7 (20),] sensitization. In this study, the normal ranges for unexposed controls in relevant concentrations of both SiNPs showed a mean SI of 0.99 ± 0.47, resulting in an SI threshold (SI + 3SD) of about 2.5 (16).

#### Measurement of cellular viability

Cellular viability of PBMC cultures were determined by fixable viability dye (FVD-780) in the total population (%) of lymphocytes that were determined by side scatter (SSC-A)/forward scatter (FSC-A) gatings using FlowJo software (FlowJo™ v10.4).

#### Measurement of immunoglobulin levels with ELISA analysis

Immunoglobulins IgM (IgM human uncoated ELISA kit, ref. 88-50620-88) and IgG (total) [IgG (total) human uncoated ELISA kit, ref. 88-50550-22] were measured in cell culture supernatant by ELISA according to manufacture protocol (Invitrogen, ThermoFisher Scientific) with detection limits at 15.6 ng/ml and 1.6ng/ml, respectively.

#### Cytokine profiling with ELISA analysis

Cytokine concentrations of granulocyte-macrophage colony-stimulating factor (GM-CSF), interferon-γ (IFN-γ), IL-1β, IL-4, IL-6, IL-12p70, IL-10, IL-13, IL-17A and tumor necrosis factor-alpha (TNF-α) were measured in cell culture supernatant according to manufacture protocol using a custom U-plex assay (Meso Scale Diagnostics, Maryland, USA) and ELISA (IL-6 and IL-13 Duoset ELISA kits, R&D systems, Minneapolis, MN, USA). Measurements were performed according to manufacturer’s instructions. Lower limits of detection (LLOD) were 0.12 pg/ml, 1.7 pg/ml, 0.15 pg/ml, 0.076 pg/ml, 9.4 pg/ml, 0.69 pg/ml, 0.14 pg/ml, 93.8 pg/ml, 2.1 pg/ml, and 0.51 pg/ml respectively.

#### Measuring *ex vivo* aggregates

Forty percent of the area of each well (of a 96-well plate)—i.e., 12 separate high-power fields per well—were imaged and evaluated (10x; EVOS). The diameter (µm) and number of aggregates across the 12 fields were measured and counted respectively, with ImageJ software version 1.53c.

#### Statistical analysis

##### Lymphocyte proliferation with CFSE assay

Unpaired two-tailed t-tests across mean ± SD SI values of silicotic patients and controls were performed separately for Ludox and NM-200, at concentrations 0.01, 0.1, 1 and 10 µg/ml. Statistical significance was defined as *, p <0.05, **, p <0.01 and ***, p <0.001.

##### Activation status of lymphocyte subsets based on CD25^+^ expression

Tukey’s multiple comparisons tests *via* two-way ANOVA was performed to determine significance of SiNP-stimulated conditions against unstimulated condition within each subject group, ^#^: significant at p<0.05 and between the two groups of subjects per exposure condition; *: p <0.05, **: p <0.01 and ***: p <0.001. ANOVA F-tests or level of significance between both groups regardless of exposure conditions are denoted by *: p <0.05 and ****: p <0.0001.

##### Immunoglobulins IgM and total IgG levels evaluated by ELISA

Tukey’s multiple comparisons tests *via* two-way ANOVA was performed to determine significance of SiNP stimulated IgM and total IgG levels against unstimulated condition within each subject group, ^#^: significant at p<0.05. Mann–Whitney two-tailed test was additionally performed to determine significance of IgM and total IgG levels between the two groups of subjects per exposure condition; *: p <0.05. ANOVA F-tests or level of significance between both groups regardless of exposure conditions are denoted by ****: p <0.0001.

##### Micro-granuloma modeling

Tukey’s multiple comparisons tests *via* two-way ANOVA was performed to determine significance in number of aggregates across SiNP concentrations against unstimulated condition within each subject group, ^#^: significant at p<0.05 and between the two groups of subjects per exposure condition; *: p <0.05, **: p <0.01 and ***: p <0.001. ANOVA F-tests or level of significance between both groups regardless of exposure conditions are denoted by ****: p <0.0001. Kruskal-Wallis test *via* one-way ANOVA test was performed to determine significance in distribution of aggregates per pair of concentrations and between both groups with significance at ****: p <0.0001.

##### Cytokine secretion levels evaluated by custom u-plex assay and ELISA

Tukey’s multiple comparisons tests *via* two-way ANOVA was performed to determine significance of cytokine levels secreted in response to SiNP concentrations against unstimulated condition within each subject group, ^#^: significant at p<0.05 and between the two groups of subjects per exposure condition; *: p <0.05, **: p <0.01 and ***: p <0.001. ANOVA F-tests or level of significance between both groups regardless of exposure conditions are denoted by *: p <0.05, **: p <0.01, ***: p <0.001 and ****: p <0.0001.

For multivariable analysis, cytokine levels in stimulated conditions, were log_10_ transformed, centered, and scaled before subgroup analyses. To investigate differences in cytokine levels between both groups, partial least squares discriminant analysis (PLS-DA) was performed. The analytics were performed using R version 4.1.2.

## Results

### 1) Subject characteristics

Blood samples were acquired from two study groups: (1) four patients diagnosed with chronic or acute silicosis (indicated as *silicosis patients*) and (2) four age-matched disease-free control subjects (indicated as *controls*) (**Table 2**). The mean age of patients and healthy controls are 46 and 42, respectively.

**Table 2:** Subject characteristics of study population upon recruitment.

	Controls				Silicosis patients				
	Control 1	Control 2	Control 3	Control 4	Silicosis 1	Silicosis 2	Silicosis 3	Silicosis 4
Industry	Desk-bound	Desk-bound	Desk-bound	Desk-bound	Artificial stone manufacturing	Brick manufacturing, sandblasting	Artificial stone manufacturing	Artificial stone manufacturing
Years of silica exposure	0 y	0 y	0 y	0 y	11 y	12 y	3 y	13 y
Smoking	Never smoked	Never smoked	Never smoked	Never smoked	Former smoker	Current smoker	Current smoker	Former smoker
Packyears (PY)	0 y	0 y	0 y	0 y	35 y	34 y	14 y	30 y
Age at blood acquisition or silicosis diagnosis	57	42	35	39	59	50	29	54
Medication	–	–	–	–	-	Methylprednisolone 1mg/day, Methotrexate 20mg 1/week	-	-
Silicosis diagnosis	No	No	No	No	Chronic Silicosis	Chronic Silicosis	Acute Silicosis	Chronic Silicosis

### 2) Lymphocyte proliferation of silicosis patients and controls

In our preliminary set of experiments, we noticed that 100 µg/ml of Ludox and NM-200 (SiNPs) was cytotoxic to PBMCs in culture, and hence this concentration was removed from further consideration (**Figure S1**).

In all controls, no significant change in SI values was observed for all tested concentrations (0.01 µg/ml up to 10 µg/ml) of both SiNPs indicating no substantial proliferation of T (CD3^+^) and/or B (CD19^+^) cells (**Table 3**).

**Table 3:** Mean stimulation index (SI) values ± standard deviation (SD) of all lymphocyte subpopulations, across 4 concentrations of SiNPs- Ludox and NM-200.

Mean SI ± SD	Controls (n=4)				Silicosis patients (n=4)			
**Ludox (µg/ml)**	0.01	0.1	1	10	0.01	0.1	1	10
CD3^+^ & CD19^+^	1.13 ± 0.42	1.13 ± 0.37	1.13 ± 0.45	0.97 ± 0.50	1.94 ± 0.67*	2.63 ± 0.28***	2.90 ± 1.57***	10.14 ± 8.50***
CD3^+^	1.01 ± 0.51	1.00 ± 0.43	1.12 ± 0.57	0.96 ± 0.52	1.51 ± 1.04	1.76 ± 0.88*	1.56 ± 1.02	3.25 ± 2.41**
CD3^+^ CD4^+^	0.95 ± 0.58	1.27 ± 0.87	1.10 ± 0.73	0.78 ± 0.51	1.25 ± 0.81	1.62 ± 0.88	1.31 ± 0.57	1.53 ± 0.59*
CD3^+^ CD8^+^	1.01 ± 0.52	1.06 ± 0.62	1.39 ± 0.74	1.22 ± 0.69	1.59 ± 0.87	1.73 ± 0.53*	1.65 ± 0.88	5.48 ± 4.76**
CD19^+^	0.89 ± 0.50	0.73 ± 0.36	0.89 ± 0.49	1.01 ± 0.79	2.70 ± 0.92***	7.42 ± 7.66**	6.11 ± 5.03***	16.29 ± 18.9**
**NM-200 (µg/ml)**	0.01	0.1	1	10	0.01	0.1	1	10
CD3^+^ & CD19^+^	1.04 ± 0.30	0.82 ± 0.34	0.96 ± 0.33	0.95 ± 0.44	1.94 ± 0.22**	3.20 ± 0.47***	2.70 ± 0.85***	4.47 ± 3.49*
CD3^+^	1.03 ± 0.48	0.86 ± 0.33	0.89 ± 0.40	0.86 ± 0.40	1.51 ± 0.65	2.10 ± 1.45	1.60 ± 0.98	1.77 ± 1.06
CD3^+^ CD4^+^	0.85 ± 0.37	0.61 ± 0.36	0.70 ± 0.42	0.62 ± 0.33	1.08 ± 0.33	1.88 ± 1.28	1.34 ± 0.58*	1.35 ± 0.55*
CD3^+^ CD8^+^	1.19 ± 0.52	1.17 ± 0.33	0.99 ± 0.33	0.88 ± 0.37	1.53 ± 0.65	1.74 ± 0.70	1.72 ± 1.36	1.65 ± 0.78*
CD19^+^	0.83 ± 0.52	0.66 ± 0.10	0.78 ± 0.62	0.89 ± 0.58	4.19 ± 5.25	7.13 ± 7.15	5.17 ± 4.12*	6.53 ± 4.61**

Unpaired two-tailed t-test was performed to determine significance with *p <0.05, **p <0.01, ***p <0.001. SI > 2.5 is indicated in red.

The mean SI values for CD3^+^ and CD19^+^ cells combined were >2.5 across all concentrations of both SiNPs except for 0.01 µg/ml, among all silicosis patients. Although no clear dose-dependent effect was noted, an increase in SI values was most pronounced at 10 µg/ml of SiNPs, across patients (**Table 3**). All patients had an SI value >2.5 at 10 µg/ml of Ludox. Likewise, in the presence of 10 µg/ml of NM-200, SI >2.5 was noted in all but one silicosis patient with values ranging from 2.46 to 10.66.

Additionally, proliferation of helper T (CD3^+^ CD4^+^) and cytotoxic T (CD3^+^ CD8^+^) cells exposed to 10 µg/ml Ludox or NM-200 was significantly higher in silicosis patients than in controls, although SI values >2.5 were only observed in CD3^+^ CD8^+^ cells when exposed to 10 µg/ml Ludox (**Table 3**).

Considering CD19^+^ cells, SI values >2.5 were found in silicosis patients across all concentrations of both SiNPs (**Table 3**). Mean SI values of CD19^+^ cells were also significantly elevated at all concentrations of Ludox and 1 and 10 µg/ml of NM-200 within the patient group, when compared with controls.

### 3) Activation of T and B lymphocyte subpopulations

**Figure 1:**
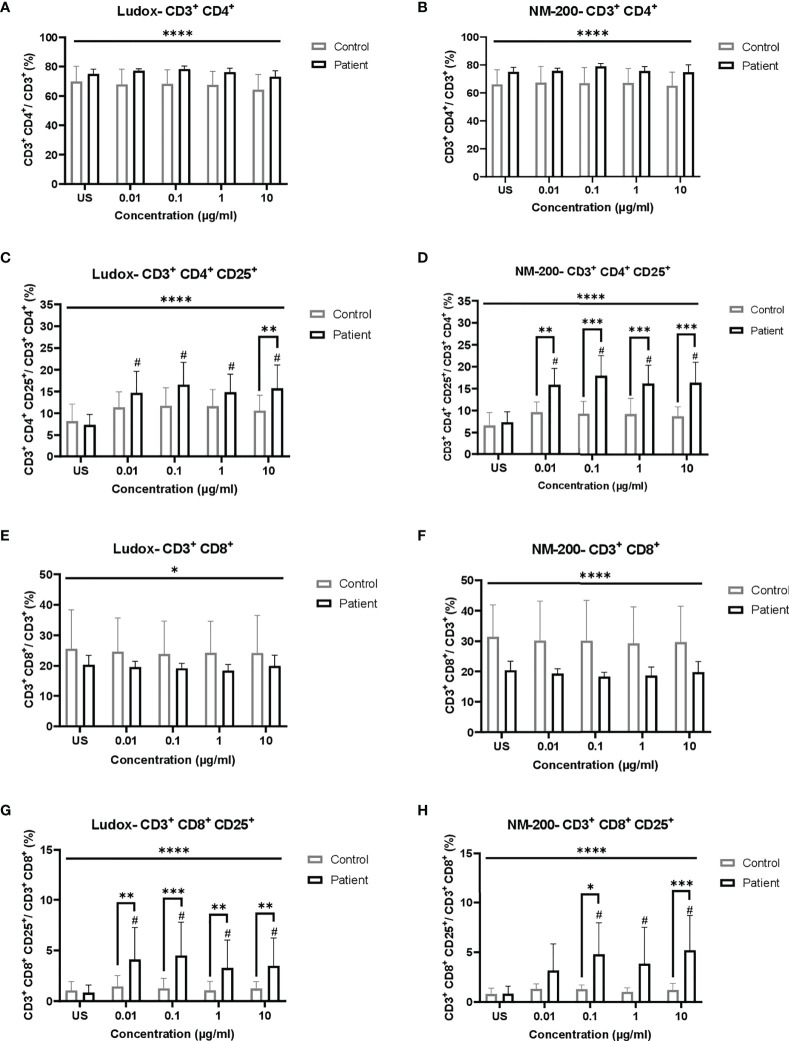
**(A–H)** Comparing the percentage of total and activated populations of CD3^+^ CD4^+^ and CD3^+^ CD8^+^ when exposed to SiNPs across both groups of individuals. Percentages of total **(A, B, E, F)** lymphocyte subpopulations of CD4^+^ **(A, B)** and CD8^+^ **(E, F)**, and activated (CD25^+^; **C, D, G, H**) subsets of CD4^+^ **(C, D)** and CD8^+^ **(G, H)**, were compared between silicotic and control groups. Tukey’s multiple comparisons tests via two-way ANOVA was performed to determine significance of SiNP-stimulated conditions against unstimulated condition within each subject group, ^#^significant at p < 0.05 and between the two groups of subjects per exposure condition; *p < 0.05, **p < 0.01 and ***p < 0.001. ANOVA F-tests or level of significance between both groups regardless of exposure conditions are denoted by *p < 0.05 and ****p < 0.0001. Non-significance (NS) within and between groups are not included. US, unstimulated.

No significant change in the percentages of CD3^+^ CD4^+^ and CD3^+^ CD8^+^ subpopulations was observed between silicosis patients and controls, per concentration of both Ludox (**Figures 1A, E**) and NM-200 (**Figures 1B, F**).

Comparing silica stimulated *vs.* unstimulated lymphocytes of controls, no increase of activated (CD25) lymphocytes was noted, while a significant increase was noted for almost all silica stimulated lymphocytes of the silicosis group (**Figures 1C, D, G, H**). Comparing the silicosis group to the controls, the CD25^+^ percentages of the CD3^+^ CD4^+^ subpopulation was significantly elevated at all concentrations of NM-200 (**Figure 1D**) but only at 10 µg/ml in the presence of Ludox (**Figure 1C**), although a significant increase was noted when compared to unstimulated population, at all concentrations of Ludox. Additionally, the percentages of CD3^+^ CD8^+^ CD25^+^ were significantly elevated in the silicotic group compared to the control group at all concentrations of Ludox (**Figure 1G**) and at 0.1 and 10 µg/ml of NM-200 (**Figure 1H**). Regardless of SiNP concentrations, significantly different percentages of T lymphocyte subpopulations and their activated subsets were also observed in silicotic patients when compared with controls (**Figures 1A–H**).

No significant differences in the percentages of CD19^+^ population between silicotic patients and controls (data not shown) was noted without stimulation.

**Figure 2:**
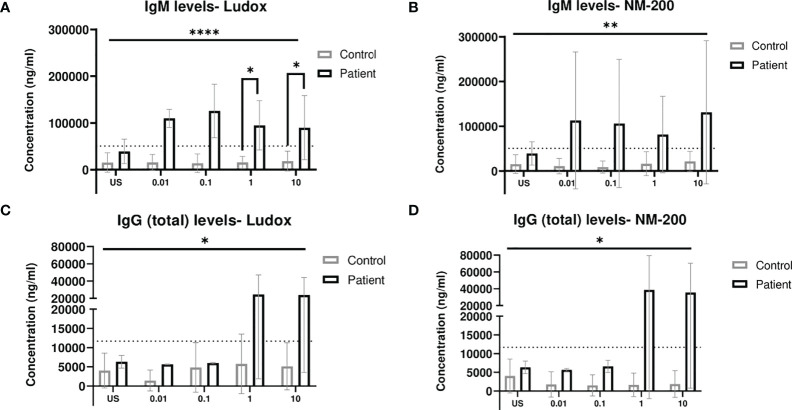
**(A–D)** Comparing IgM and total IgG release when exposed to SiNPs on Day 7. IgM **(A, B)** and (total) IgG **(C, D)** levels measured in culture supernatant on day 7, were compared between silicotic patients and controls groups. Tukey’s multiple comparisons tests via two-way ANOVA was performed to determine significance of SiNP stimulated IgM and total IgG levels against unstimulated condition within each subject group. Mann–Whitney two-tailed test was additionally performed to determine significance of IgM and total IgG levels between the two groups of subjects per exposure condition; *p < 0.05. ANOVA F-tests or level of significance between both groups regardless of exposure conditions are denoted by ****p < 0.0001. Non-significance (NS) within and between groups are not included. The dotted line represents cut-off determined based on Mean + 2SD of US concentrations in group of controls. US, unstimulated. **p<0.01.

Significantly increased IgM levels were observed within patients, at 1 and 10 µg/ml of Ludox (**Figure 2A**) but not for NM-200 (**Figure 2B**) stimulation, due to large variability. Despite this variability observed within the groups of controls and patients, IgM levels were consistently elevated at all concentrations of both SiNPs, across patients when compared with controls.

The IgG (total) levels in the supernatants of both controls and patients were also assessed with Ludox (**Figure 2C**) and NM-200 (**Figure 2D**) stimulation. No change in total IgG levels of controls were noted across all concentrations of both SiNPs. Overall, the levels of total IgG release with both Ludox and NM-200 stimulation were significantly elevated in patients when compared to controls.

### 4) Micro-granuloma modeling of SiNP-induced response

PBMC cultures were maintained up to 7 days post-exposure and cellular aggregation began around day 3 with different rates of development over time, in both SiNP-stimulated as well as unstimulated conditions.

**Figure 3:**
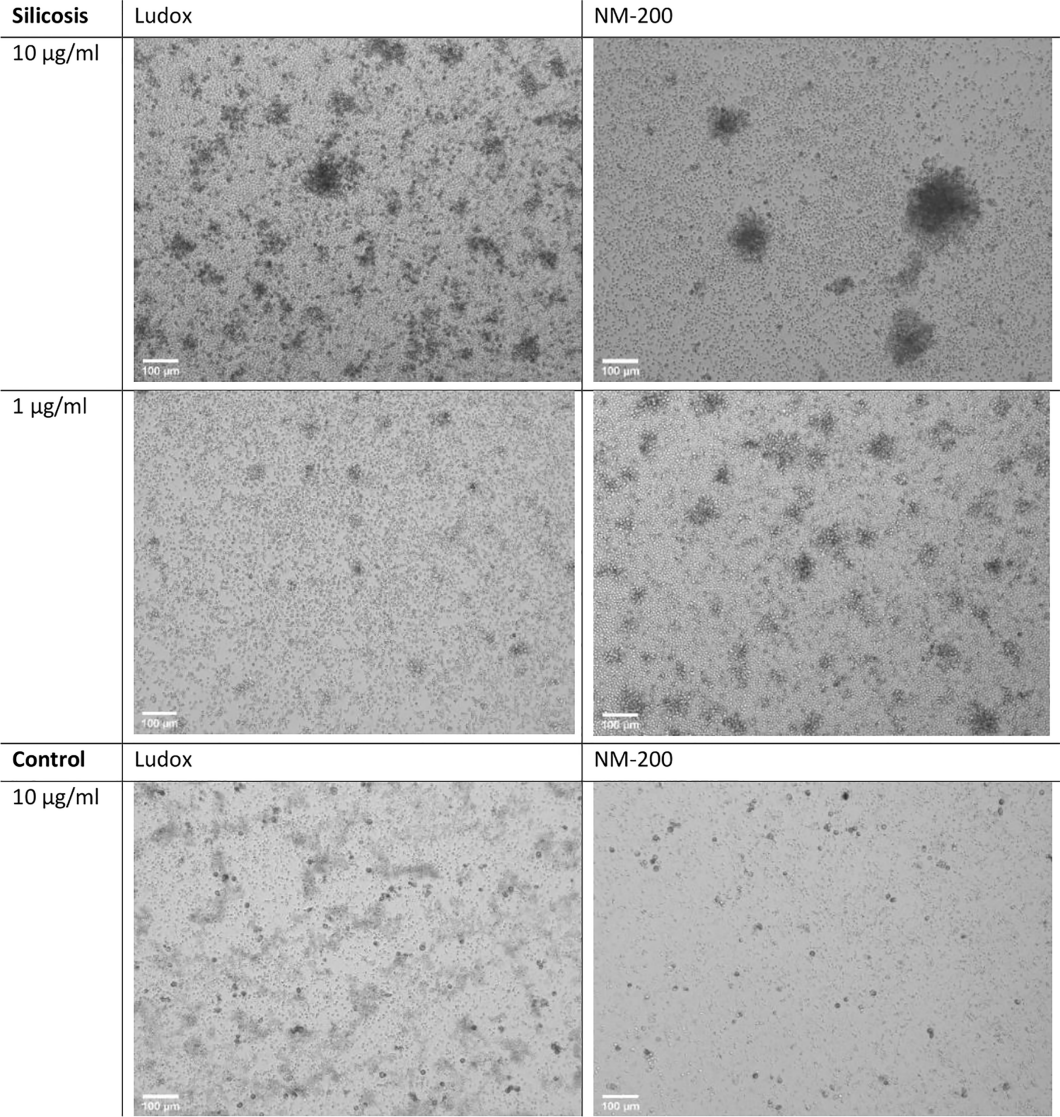
Microstructure of developing aggregates. PBMCs from silicotic patients and controls, were stimulated with Ludox and NM-200, with a starting concentration of 10 µg/ml. Representative images of day 7 (10x, EVOS) from 1 out of 12 images taken per well (replicate) from a single individual per group are included, scale bar: 100 µm.

**Figure 4:**
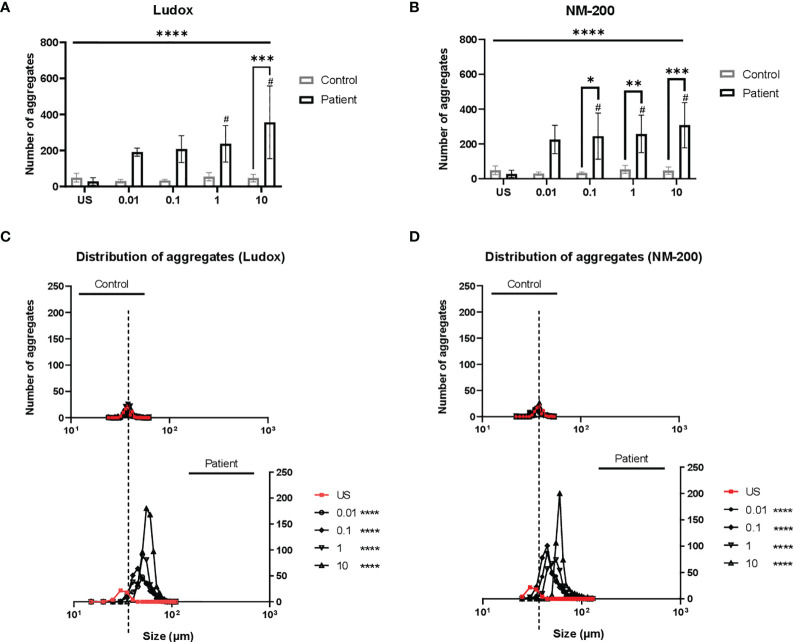
**(A–D)** Distribution of aggregates in patients from controls. On day 7, the diameter (µm) and number of aggregates were measured and counted across two replicates. The mean number of aggregates at concentrations 0.01 to 10 µg/ml of Ludox **(A)** and NM-200 **(B)** within patients were compared with the controls. Differences in frequency distribution (accounts for size and number of aggregates) were additionally compared between silicotic patients and controls at all exposure conditions for Ludox **(C)** and NM-200 **(D)**. Tukey’s multiple comparisons tests via two-way ANOVA was performed to determine significance in number of aggregates across SiNP concentrations against unstimulated condition within each subject group, ^#^significant at p < 0.05 and between the two groups of subjects per exposure condition; *p < 0.05, **p < 0.01 and ***p < 0.001. ANOVA F-tests or level of significance between both groups regardless of exposure conditions are denoted by ****p < 0.0001. Kruskal-Wallis test via one-way ANOVA test was performed to determine significance in distribution of aggregates per pair of concentrations and between both groups with significance at ****p < 0.0001. Non-significance (NS) within and between groups are not included. US, unstimulated. NS- non-significance.

By day 7, a consistently lower number of aggregates were counted in controls when compared to silicotic patients, while larger aggregates were specifically observed within silicotic individuals (**Figure 3**). After stimulation with silica, the number and size of aggregates did not significantly change in controls (**Figure 4**).

A significant increase in number (**Figures 4A, B**) and size (rightward shift in **Figures 4C, D**) of the aggregates in PBMCs of silicosis patients was seen, as opposed to controls.

**Figure 5:**
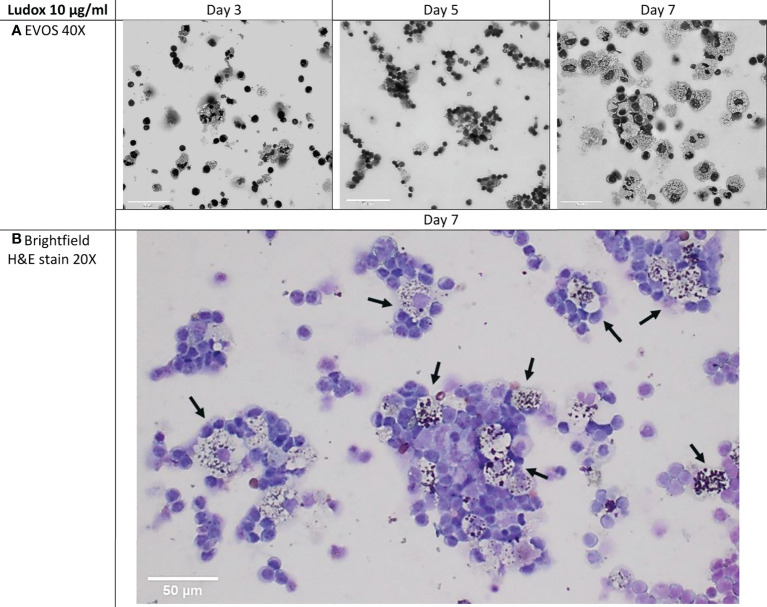
**(A, B)** Cellular makeup of developing aggregates over select time-points. Cytospins of developing granulomatous aggregates that were stimulated with 10 µg/ml of Ludox, were H&E stained on days 3, 5 and 7 within the silicotic patient group. EVOS images were captured at 40x, scale bar: 50µm **(A)** and compared against brightfield imaging at 20x, scale bar: 50µm **(B)**. By day 7, loosely organized granulomatous structures were retained and most of the macrophages found within these structures contained Ludox silica particles (black arrows) and were surrounded by layers of lymphocytes (Brightfield, 20x). H&E, hematoxylin and eosin.

At days 3, 5 and 7 the cellular makeup of the developing structures was assessed by H&E staining. The multicellular aggregates encompassing lymphocytes and macrophages, were prominent from Day 5 onwards, with increment in size and lymphocyte-like cellular recruitment observed over time. The developing granulomatous structures were also retained by the end of the exposure with marked lymphocyte aggregation around (foamy) silica-laden macrophages. (**Figures 5A, B**).

### 5) Cytokine secretion profiles of SiNP stimulated PBMCs

The extent of Th1, Th2 and Th17 pro-inflammatory cytokines and Th2 anti-inflammatory cytokines released in silicotic and healthy subjects were evaluated before further examination with multivariate analysis to identify the key cytokines that contribute towards silicosis.

#### 5I) Comparing cytokine levels between silicosis patients and controls on day 7

**Figure 6:**
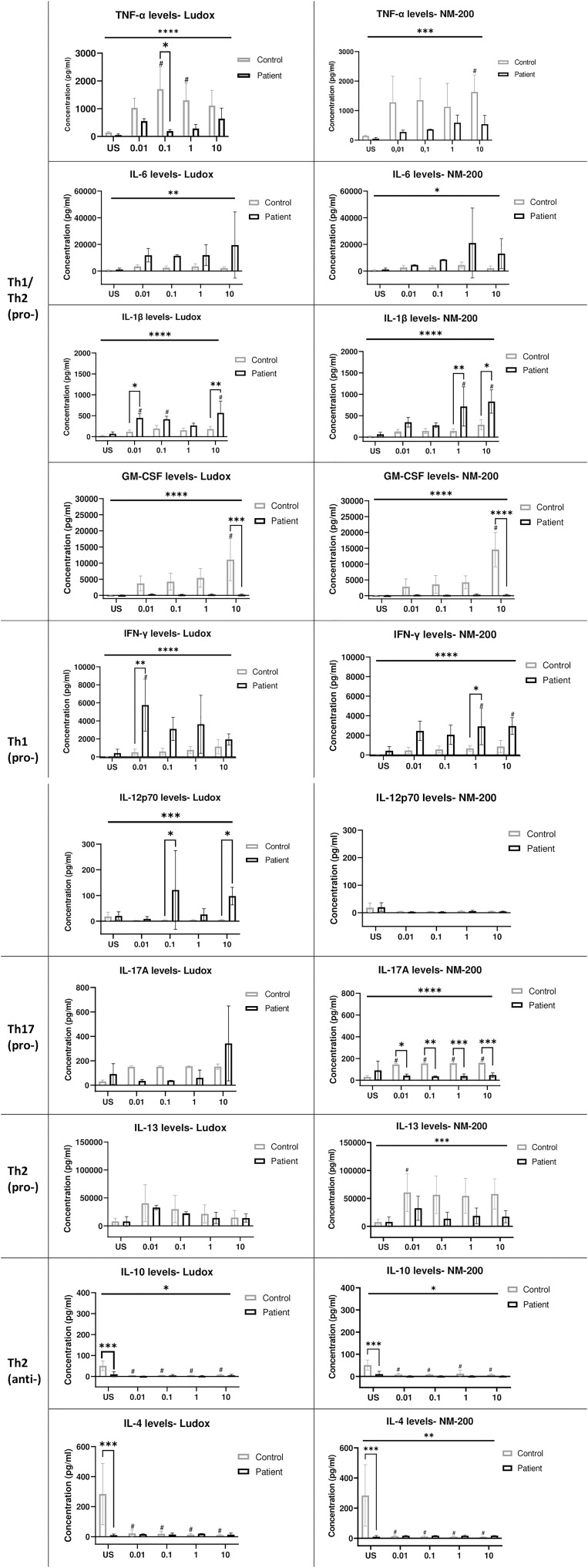
Cytokine secretion profiles of silicotic and healthy subjects on day 7. The cytokine mediated immune response in silicosis patients and controls were determined on day 7 with an ELISA for pro-inflammatory (pro-) cytokines generated by multiple (Th1/ Th2) cells: TNF-α, IL-6, IL-1β, GM-CSF; Th1 cells: IFN-γ, IL-12p70; Th17 cells: IL-17A and Th2 cells: IL-13. Anti-inflammatory (anti-) Th2 cytokines: IL-10 and IL-4 were also evaluated between both groups. Tukey’s multiple comparisons tests *via* two-way ANOVA was performed to determine significance of cytokine levels secreted in response to SiNP concentrations against unstimulated condition within each subject group, ^#^significant at p < 0.05 and between the two groups of subjects per exposure condition; *p < 0.05, **p < 0.01 and ***p < 0.001. ANOVA F-tests or level of significance between both groups regardless of exposure conditions are denoted by *p < 0.05, **p < 0.01, ***p < 0.001 and ****p < 0.0001. Non-significance (NS) within and between groups are not included. US, Unstimulated. NS, non-significance.

The cytokine release or expression profiles across different concentrations of SiNPs were evaluated on day 7 (**Figure 6**). Without a stimulus, cytokine levels did not differ significantly between both patients and controls, except for IFN-γ and IL-17A (albeit non-significant) for which the level in patients was higher and for IL-4 and IL-10 which was higher in controls. Comparing Ludox and NM-200 stimulation, similar trends across patients are observed with elevated or decreased release of cytokines except for IL-12p70 and IL-17A.

When comparing SiNP-stimulated PBMCs between silicosis patients and controls, some specific differences were noted:

Significantly increased release of pro-inflammatory cytokines IL-6, IL-1β, IFN-γ and IL-12p70 (the latter only with Ludox stimulation) was observed in silicosis patients, compared to controls, while significantly lower levels of TNF-α, GM-CSF, IL-13 and IL-17A (the latter two only with NM-200 stimulation) were found in patients compared to controls.

IL-12p70 was significantly elevated in patients upon Ludox stimulation while no change in IL-12p70 levels was noted when stimulated with NM-200. IL-17A levels were increased in patients at highest concentrations of 10 µg/ml Ludox, whereas a significant decrease in levels of IL-17A were observed in patients when compared with controls upon NM-200 stimulation.

Additionally, no difference in the release of anti-inflammatory cytokines IL-10 and IL-4 was noted between the groups of individuals, when stimulated by either SiNPs.

#### 5II) Fingerprint of disease based on cytokine levels—PLS-DA

**Figure 7:**
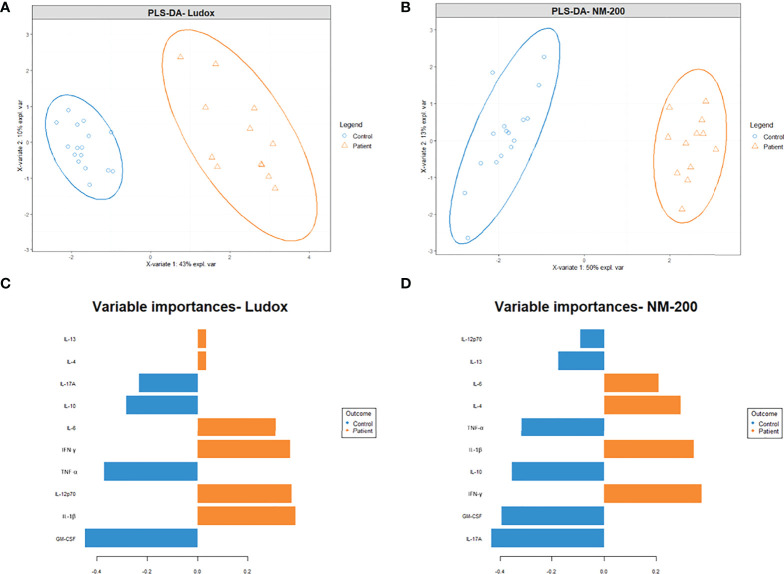
**(A–D)** Projection of multi-cytokine data to identify for differences in cytokine profiles between patients and controls. A partial least squares discriminant analysis (PLS-DA) plot was made with the log_10_ transformed, centered, and scaled multi-cytokine data between patients and controls on day 7. Strong differing profiles between patients and controls were noted with Ludox **(A)** and NM-200 **(B)** stimulation. A variable/ cytokine importance plot for Ludox **(C)** and NM-200 **(D)** stimulation were also made to show the degree at which individual cytokines contribute towards the differences in the outcome/ groups.

To clarify the cytokine secretion profiles that could be associated with silicosis, the multi-cytokine data per group of patients and controls, were explored with partial least squares discriminant analysis (PLS-DA, **Figure 7**). Firstly, both controls and patients’ groups were clearly discriminated upon stimulation with Ludox (**Figure 7A**) and NM-200 (**Figure 7B**).

Secondly, to identify which cytokines contributed to this distinguishment, per type of SiNP, a cytokine (variable) importance plot was made to quantify the contribution of each cytokine to the outcome (control versus patient) (**Figures 7C, D**). Based on the variable importance plot, IL-1β, IL-12p70, IFN-γ and IL-6 are strongly upregulated in silicotic individuals upon Ludox stimulation while IFN-γ, IL-1β, IL-4 and IL-6 are strongly upregulated upon NM-200 stimulation. At the same time, GM-CSF, TNF-α, IL-10 and IL-17A are downregulated in patients when stimulated with both Ludox and NM-200.

## Discussion

In this study, we focused on characterizing the influence of silica particles – more specifically nanoparticles (SiNPs) – on immune cells of silicosis patients and controls, based on the proliferative capacity and activation status of select lymphocyte subsets. In addition, granulomatous response to silica was evaluated by comparing the extent of aggregate formation with the cytokine secretion profiles. We showed that PBMCs of silicosis patients respond differently to SiNPs than those of healthy subjects. Firstly, the SI values based on lymphoproliferative test, CFSE assay, was elevated in silicotic patients as opposed to controls when the B cells (CD19^+^) population was accounted for. Secondly, subsets of regulatory T cells—CD3^+^ CD4^+^ CD25^+^ and CD3^+^ CD8^+^ CD25^+^—as well as immunoglobulins IgM and IgG, were also significantly increased in PBMCs of patients. Thirdly, the number and the size of granulomatous aggregates formed were higher with SiNPs stimulation in patients compared to controls. Lastly, cytokines known to be associated with silicosis were also differentially changed in the medium of SiNP-stimulated PBMCs of patients compared to controls.

The response of adaptive immunity components to silica particles is related to the cascade of activities mediated by the interaction with cells involved in innate immunity, particularly macrophages (21), T cells and B cells (22). Various studies indicate that silica does not act as an antigen (23) but that it supports the increase of antigen-presenting properties of macrophages (24) instead. However, direct interaction of T cells or even B cells with silica cannot be excluded (21).

To determine the extent of cellular interactions involved, we first focused on assessing the lymphoproliferative capacity of CD3^+^ T cells and its subsets CD4^+^ T helper, CD8^+^ cytotoxic T as well as CD19^+^ B cells, when stimulated with SiNPs. A clear dose-dependent and positive (SI > 2.5) response in SI values with increasing concentrations of both Ludox and NM-200 were only observed with CD3^+^ and CD19^+^ combined and with CD19^+^ cells only. At the same time, a lack of positive response in T cell subsets of patients was also noted at most concentrations of SiNPs. On this basis, we speculate that B cells may play a prominent role in the pathogenesis of silicosis. Similar to our observations, animal experimental studies have identified that B cells, specifically B-1 lymphocytes, respond to inorganic particles like silica by migrating towards the inflammatory site, engaging in phagocytosis alongside macrophages to engulf particles and partake in granuloma formation by organizing inorganic particle-induced cellular aggregation into three-dimensional granulomatous structures (25).

Despite the lack of T cell proliferation, a consistent and significantly increased percentage of activated T cells [CD25^+^, (26)] was observed in patients when compared with the controls, in our study. One of the early-recognized events of T cell receptor (TCR)-dependent T cell activation is the increase in the cytoplasmic [Ca^2+^] level (27). Silica instillation studies in rats have shown that anti-CD3–induced elevation in [Ca^2+^] is exaggerated in T cells at 4 days and 4 weeks after silica exposure, confirming that chronic exposure to silica affects the TCR-mediated signaling in T cells (28). Comparatively, in human studies; activation of T cells in silicotic patients due to the recurrent and chronic direct contact between T cells and silica, has also been reported (29).

The lack of T cell proliferation in PBMCs of patients suggests limited interaction and recognition of antigen fragments on B cell surfaces, by T cells (30). Similarly, Vis and colleagues also observed minimal proliferation of T cell subsets while markers of T-cell activation were increased (12). They also proposed that the interaction between T-cell independent antigen – silica nanoparticles <10nm, and T cells does not require antigen presenting cells (APCs) for cross-linking interactions (12). T-cell independent antigens are also capable of non-specific activation of both immature—commonly termed activated B cells (31)—and mature B cells (32), accompanied by the generation of mostly IgM. One can postulate that the observed T-cell activation and proliferation of B cells in our study, is not T-cell mediated, and so SiNPs could most likely function as a T-cell independent antigen.

T cell activation upon the recognition of antigenic peptides presented by APCs *via* major histocompatibility complex MHC II complex (T-cell dependent), would non-specifically stimulate T cell proliferation (30). If SiNPs function as T-cell dependent antigens to activate and stimulate T and B cells simultaneously, then isotype class switching of immunoglobulins (Ig) with a potent IgG response would be anticipated (30). Although IgG levels were elevated in the patients at higher SiNPs concentrations, we only observed significantly increased secretion of IgM when compared to the controls, in our study. This firstly highlights the presence of Ig producing-activated B cells (CD19^+^) or plasma cells (not CD19^+^) and secondly suggests that SiNPs might not be capable of inducing B cell isotype switching or affinity maturation which is in fact dependent on the involvement of T cells (30). Our findings on the increased prevalence of elevated IgM and IgG levels supports the impact of B cell hyperactivity observed in silicosis and the notion of direct polyclonal stimulation of B cells by silica particles (33).

In a previous animal experimental silicosis study, significantly increased serum IgG levels were observed at all timepoints from 2 to 5 months, whereas significantly elevated serum IgM levels were only noted at the 4^th^ and 5^th^ month (34). Likewise, patients with silicosis exhibited increase in serum IgG levels which were also more pronounced than the increase in serum IgM levels (33). The release of IgM and IgG has however, not been shown to be increased at earlier timepoints i.e., early stages of silicosis (34), and serum Ig levels in humans were not evaluated immediately following acute silica exposure (33). As such, a knowledge gap still exists on the relationship between humoral immunity and progression of silicosis. At the same time, we cannot disregard the fact that IgM functions as the first line of host defense (35) in the presence of foreign antigens and, as observed in our study, elevated IgM levels were noted between 4 days (**Figure S2**) and 7 days of exposure.

To avoid bias in data based on *a priori* selection of biological variables, we knowingly accounted for a balance between Th1, Th2 and Th17 cytokines in our study. A dominant Th1 and to a smaller extent Th17 response are typically observed in the early inflammatory stage of silicosis while an elevated Th2 response is noted during the later development of fibrosis in humans and *in vivo* (36). PLS-DA of silicosis patients in our study, further documented the connections between the outcome of silicosis and cytokine predictors: Th1/Th2 pro-inflammatory IL-6, IL-1β and IFN-γ, which were significantly elevated in silicosis patients when compared to controls. We also posit that the increasing expression of IL-6, IL-1β, IFN-γ and GM-CSF within silicotic individuals could be induced by early micro-granuloma development since it is a key mediator during acute phase response of silicosis (37).

As inflammatory cytokines IL-6, IL-1β, TNF-α are also potent inducers of GM-CSF (38), it is not surprising that these cytokines are strongly associated with each other and act as predictors for silicosis patients. At the same time, B cells are the major source of IL-6 (39) and can also modulate the immune response with the secretion of TNF-α, IL-6 and GM-CSF to assist in disease progression (40). Previous findings suggest that patients with silicosis exhibited higher IL-6 serum levels than in unexposed healthy individuals (41), which is similar to our observations. IL-6 also has important roles in T and B cell activation and also causes CD4^+^ and CD8^+^ T cells and regulatory T cells to trigger the maturation of B cells downstream of the signaling cascade (42). IFN-γ has also been implicated in reducing total IgG levels secreted by B cells in humans and additionally has the ability to antagonize the synthesis of IgG1 by IL-6 (43); which aligns with our findings of lower IgG levels secreted. As such, a link may be drawn between elevated levels of B cells found within the silicotic group of our study and the predicted inflammatory signature based on multivariable analysis performed.

IL-1β has also been implicated in the pathophysiology of silicosis in both humans and animal experimental studies (44). Additionally, IL-1β is also suggested to be a vital trigger in determining the silica-induced secretion or activation of other cytokines in co-culture systems made up of monocytes/macrophages and endothelial cells (45). In mice, IL-1β levels in bronchoalveolar lavage fluid (BALF) remained significantly elevated upon silica stimulation (46) and IL-1β-deficient mice are reportedly protected from silica-induced lung inflammation, as determined by the lack of silicotic lesions and inflammation observed in histological sections (47). In addition, primary human monocyte derived macrophages (MDMs) and macrophage-like cell line, THP-1, also secreted mature IL-1β on stimulation with silica (48). Our findings thus concede with previous observations, since IL-1β levels remain elevated and significant in silicotic patients; especially at the highest concentration of 10 µg/ml of both SiNPs when compared with controls.

GM-CSF is essential for proliferation, chemotaxis and survival of monocytes as well as its differentiation to macrophages (49). Likewise, in our study, the increasing GM-CSF levels observed over time from day 4 (**Figure S3**) to 7, suggests continued monocyte-macrophage maturation in silicosis patients, whereas this process might have reached a state of resolution before the end of the exposure period for healthy controls. In fact, by day 7, lymphocytes of silicotic patients had formed more and larger aggregates around silica-laden macrophages resembling micro-granulomas when compared with the controls. It is thus evident that contact between these two cell types is required for the initial stages of granuloma formation, and such cellular interactions could possibly be mediated by matured or activated macrophages because of GM-CSF regulation. Although the granulomatous structures formed resemble early granuloma formation, they cannot be unequivocally regarded as a well-developed granuloma.

IFN-γ is predominantly secreted by activated CD3^+^ CD4^+^ and CD3^+^ CD8^+^ T cells to further activate and promote macrophages to increase phagocytosis, intracellular killing of pathogens such as bacteria and secrete higher levels of pro-inflammatory cytokines such as TNF-α (29). Previously, co-cultivation of lymphocytes and macrophages from silicotic nodules has shown increased IFN-γ production in rats (50). Additionally, up-regulation of IFN-γ has been detected in silica-exposed workers (51). In our study and at the end of the exposure period, IFN-γ levels remained higher in silicosis patients highlighting a possible interaction between activated T cells and matured macrophages. The IFN-γ/IL-13 expression ratio was also reportedly higher in our silicotic patients compared to controls and is consistent with a classically activated (M1) macrophage polarization balance, considering that IL-13 is also recognized as a proximal regulator of alternatively activated (M2) macrophage polarization (52). Although it remains unclear if IL-13 levels were either elevated among controls or were in fact suppressed within silicotic patients, the reduced levels could suggest inadequacy in suppressing inflammatory signaling of cytokines like IL-1β and IFN-γ – further attenuating fibrosis.

Studies have reported that, on one hand, TNF-α plasma levels were significantly higher in silicotic patients when compared with controls (53), while on the other hand, although no significant differences were observed, serum levels of TNF-α were the lowest in the complicated silicosis group when compared with the healthy controls group (37). As such the dual role of TNF-α as both an inflammatory and fibrogenic cytokine (44) remains equivocal. Nonetheless, the increasing TNF-α levels observed over time, in the silicotic group from day 4 (**Figure S3**) of our study, could be an indicator of its early pro-inflammatory function in disease development. Alternatively, the reduced TNF-α levels observed in silicotic patients on day 7, could be a consequence of counterbalance between Th1 pro-inflammatory cytokines, since TNF-α is also required for the development of silica-induced fibrosis (54).

Contrasting opinions on the exact roles of Th2 cytokines IL-10 and IL-4 in the course of silicosis development have thus far been proposed. In experimental silicosis, less intense fibrosis in response to silica particles was observed in IL-10 deficient mice when compared with wild-type mice (55), while the inversed effect has also been observed in the livers of IL-10 deficient mice (56). In this capacity, it is possible that IL-10 downregulation as observed in our study, may limit pulmonary inflammation and subsequently control the extent of collagen accumulation observed in the inflammatory stages of silicosis (57). Similarly with IL-4, abundance of IL-4 signaling following quartz instillation (57) as well as decreased IL-4 mRNA expression in lymph nodes of rats with silicosis (58) have been reported. Overall, the downregulation of IL-13, IL-10 and to a lesser extent IL-4 reported in this study, strongly supports the concept of their pro-fibrotic roles in silica-induced fibrosis rather than the pro-inflammatory roles of their counterparts, IL-1β, IL-6 and IFN-γ, during the early inflammatory response stage.

It is necessary to underline those cytokines like TNF-α and IL-1β, are also highly secreted by macrophages in response to silica particles, and therefore can also participate in SiNP induced perpetuation of inflammation, in turn sustaining the increase of lymphocytes and interaction with macrophages observed. Although we did not conduct experiments to identify possible specific proteins responsible for macrophage maturation and migration to SiNPs, several studies that did so, support our findings with an added focus on viability and proliferation of macrophages when exposed to micro- and nano- silica (59) and provided evidences of macrophage secretome induction by Si NP exposure (60). We thus can speculate that both mechanisms may co-exist i.e., T-cell independent role of SiNPs and function of macrophages as APCs since we also observed phagocytosis of SiNPs by cells resembling macrophages/mature monocytes (**Figure 5**).

This mirrors human silicosis, since phagocytosis and release of silica particles by alveolar macrophages in the lungs drives disease progression (6) and is also mediated by the secretion of IFN- γ that promotes macrophages to increase phagocytosis (29). Lung sections of silica-treated 6 weeks old animals also presented nodular aggregation of B lymphocytes exhibiting surface membrane staining for IgM, around blood vessels and bronchioles (61), further supporting our findings that B cells not only mediate disease progression, but they are also implicated in granuloma development during the early development stage of silicosis. Therefore, it would also be of interest to explore macrophages’ function as APCs alongside B cell function in future studies with the use of PBMCs.

It is well established that crystalline silica is much more reactive and more often linked to silicosis due to its widespread availability in natural forms such as quartz. Based on how amorphous silica is produced and their likely enhanced ability to penetrate intracellular targets in the lung (62), there is also good reason to believe that amorphous nano silica can be equally hazardous due to their very diverse entity when compared with the crystallinity of crystalline silica (63).

Therefore, the differences reported in amorphous SiNP effects on activated lymphocyte subsets and aggregate sizes and mean numbers; although utilized at the same concentrations could be related to differences in primary particle size, size distribution, method of preparation i.e., probe sonication versus vortex; and their respective surface properties (62). In fact, amorphous SiNPs with same specific area but different surface chemistry can be easily prepared, further adding to the complexity on deciphering the pathogenic activity of silica, whether crystalline or amorphous (64). Regardless, our observations highlight the importance of individual effects of amorphous SiNPs that may initially be presumed to be similar in both physico-chemical and toxicological properties, which could warrant further investigation on effects of silica burden on PBMCs.

### Strengths and limitations of our study

Monitoring stimuli-specific lymphocyte response to mitogens, *Candida albicans*, tetanus and even beryllium to measure immune reactivity associated with immunosuppression, autoreactivity and hypersensitivity have been well described in several studies (65). However, it remains unknown if lymphocyte proliferation can remain equally specific and sensitive against novel compounds such as silica particles. Moreover, phenotypical characterization of proliferating cells using cell surface markers have mostly been limited to CD3^+^ T cells. The inclusion of CD19^+^ B cells in our study addressed both of these shortcomings, and as a result, the lymphoproliferative capacity of nano-silica stimulated CD3^+^ and CD19^+^ combined, and more specifically CD19^+^ cells only; in silicotic patients, is reported for the first time.

Additionally, although silicotic nodules with concentric fibrosis are typically observed during the chronic stages of silicosis – which is also the most frequent timepoint of diagnosis; early cellular lesions resembling granulomatous structures consisting of silica dust-laden macrophages have also been reported in silicotic patients (1). Our findings thus support this notion, since silica-laden macrophages surrounded by a core of lymphocytes in early-stage granulomas were noted.

Although our study has thus far attempted to holistically address both cellular interactions and micro-granuloma modeling in silica-stimulated conditions, there are still several limitations that ought to be addressed. The major limitations of this study are the small sample size due to the rarity of the disease. It is also likely that the cellular environment has a strong influence on how silica particles are presented to and recognized by circulating cells, and such differences can also be modulated and investigated *in vitro/ex vivo*. Though the role of MDMs in a multicellular environment were not fully investigated in this study, its function as APCs and involvement in secretion of cytokines when exposed to inflammatory stimuli, should not be disregarded. Lastly, the differences in the impact of crystalline silica, such as Min-U-Sil 5, an α-quartz that is commonly used in animal experimental silicosis studies (34), as opposed to amorphous silica like Ludox and NM-200 used in this study, have not been explored in immune cells of silicotic patients.

## Conclusion

Although the interplay of cellular mechanisms and resulting consequences in the development of chronic or late-stage silicosis (61) have been studied extensively, the events that prelude its development, still remain elusive. Inconsistency in modeling different pathophysiological stages i.e., from early to late (normal, inflammatory, progressive to fibrotic) in experimental silicosis (4), limited cross-validation in human studies that lack comparison with silicosis patients and the relevance of experimental models for humans, are some of the contributing factors. Moreover, there is no clear evidence on which cellular types are (in)directly regulating silicosis development.

Thus far, the proliferative capacity of B-cells stimulated by inorganic compounds has not been investigated and our study is the first of its kind to report such findings. This is also the first study that has shown that it is possible to generate micro-granulomas in response to a novel compound such as silica. Within the silicotic group, the *ex vivo* model also successfully recapitulated the inflammatory stage of silicosis development in terms of Ig levels with elevated IgM rather than total IgG levels observed by day 7 and with cytokines’ secretion, wherein a predominant Th1 pro-inflammatory signature despite the counterbalance of Th1 cytokines’ release was observed after silica exposure.

To conclude, this model has prospects to provide insights into host-antigen interactions at various stages of granuloma development in silicosis. On a cellular level, investigating cell-cell interactions that contribute to its maturation and maintenance after a single exposure can address the current limitations of animal experimental silicosis studies. The induction of B cells proliferation or identifying the exact pathways that mediate its proliferation can also be a future consideration for treatment or management of silicosis.

## Data availability statement

The raw data supporting the conclusions of this article will be made available by the authors, without undue reservation.

## Ethics statement

The studies involving human participants were reviewed and approved by Ethics Committee Research UZ/KU Leuven (S61777). The patients/participants provided their written informed consent to participate in this study.

## Author contributions

Conceptualization: NG, SR, and PH; investigation: NG; Methodology: NG; writing- original draft preparation: NG; writing- review and editing: NG, SR, and PH; visualization: NG; supervision: SR and PH; funding acquisition: SR and PH. All authors contributed to the article and approved the submitted version.

## Funding

This research was funded by KU Leuven Internal Funding C2 (C24/18/085).

## Acknowledgments

The authors would like to thank 1) senior lab technician Jonathan Cremer for his assistance and valuable knowledge in flow cytometric analysis of samples and 2) Prof. Bart Vanaudenaerde for allowing us to use his equipment: EVOS cell imaging system.

## Conflict of interest

The authors declare that the research was conducted in the absence of any commercial or financial relationships that could be construed as a potential conflict of interest.

## Publisher’s note

All claims expressed in this article are solely those of the authors and do not necessarily represent those of their affiliated organizations, or those of the publisher, the editors and the reviewers. Any product that may be evaluated in this article, or claim that may be made by its manufacturer, is not guaranteed or endorsed by the publisher.

## Abbreviations

CFSE, Carboxyfluorescein succinimidyl ester; COPD, chronic obstructive pulmonary disease; Ig: immunoglobulin; IFN-γ: interferon-gamma; IL-1β, interleukin-1β; MDMs, monocyte-derived macrophages; PLS-DA, partial least squares discriminant analysis; PBMCs: Peripheral blood mononuclear cells; SiNPs, silica nanoparticles; sIL-2R, soluble interleukin-2 receptor; SI, stimulation index; TGF-β, transforming growth factor beta; Th1 and Th2, T helper type 1 and 2.
