# Supplementary material for: Differential immunological effects of silica nanoparticles on peripheral blood mononuclear cells of silicosis patients and controls
Source: Front Immunol. 2022 Oct 13;13:1025028. doi: 10.3389/fimmu.2022.1025028 (PMC9606771; doi:10.3389/fimmu.2022.1025028)

**Supplementary/Annex:**

Figure S1: Dose-dependent effect on viability of cells in culture

Mean ±SD percentage (%) change in viability (FVD-780) of cells stimulated with 0.01 to 100 µg/ml of SiNPs: Ludox and NM-200, with respect to control. Viability threshold is set at 75% and SiNP concentrations with a viability below 75%, are therefore removed from consideration.

|  |  |
| --- | --- |

Figure S2: Comparing the release of immunoglobulins IgM and total IgG when exposed to SiNPs on Day 4

IgM (A-B) and (total) IgG (C-D) levels measured in culture supernatant on day 4, were compared between silicotic patients and controls groups.

Tukey’s multiple comparisons tests via two-way ANOVA was performed to determine significance of SiNP-stimulated IgM and total IgG levels against unstimulated condition within the control and patient groups. Mann–Whitney two-tailed test was performed to determine significance of IgM and IgG levels between both groups at respective timepoints. #: significant at p<0.05 and between groups; *: p <0.05, **: p <0.01, ***: p <0.001. Non-significance (NS) within and between groups are not included. The dotted line represents cut-off determined based on Mean + 2SD of US concentrations in group of controls. Legend: US: unstimulated

| A) |  | B) |  |
| --- | --- | --- | --- |
| C) |  | D) |  |

| **Th1/**  **Th2**  **(pro-)** |  |  |
| --- | --- | --- |
|  |  |  |
|  |  |  |
|  |  |  |
| **Th1**  **(pro-)** |  |  |
|  |  |  |
| **Th17**  **(pro-)** |  |  |
| **Th2**  **(pro-)** |  |  |
| **Th2**  **(anti-)** |  |  |
|  |  |  |

Figure S3: Cytokine secretion profiles of patients and controls on day 4

The cytokine mediated immune response in silicosis patients and controls were determined on day 4 with an ELISA for pro-inflammatory (pro-) cytokines generated by multiple (Th1/ Th2) cells: TNF-α, IL-6, IL-1β, GM-CSF; Th1 cells: IFN-γ, IL-12p70; Th17 cells: IL-17A and Th2 cells: IL-13. Anti-inflammatory (anti-) Th2 cytokines: IL-10 and IL-4 were also evaluated between both groups.

Tukey’s multiple comparisons tests via two-way ANOVA was performed to determine significance of SiNP-stimulated conditions against unstimulated condition within each group, ^#^: significant at p<0.05 and between groups; *: p <0.05, **: p <0.01. Non-significance (NS) within and between groups are not included. Legend: US: Unstimulated

**Supplementary methodology:**

Figure S4: Gating strategy to measure proliferation of CFSE labelled PBMCs

Following the 7^th^ day of incubation, CFSE labelled cell suspensions were collected and stained with an antibody cocktail of FVD-780, AF700 anti-CD3 (UCHT1), APC anti-CD4 (RPA-T4), e450 anti-CD8 (RPA-T8) and PerCP-Cy5.5 anti-CD19. Legend: FCS: forward scatter, SSC: sideward scatter, -A: area, -H: height, -W: width, FVD780: fixable viability dye 780, CFSE: Carboxyfluorescein succinimidyl ester, CFSE+: undivided percentage of population, CFSE-: divided percentage of population, AF700: Alexa Fluor 700, APC: allophycocyanin, e450: eFluor 450, PerCP-Cy5.5: peridinin chlorophyll Cy5.5


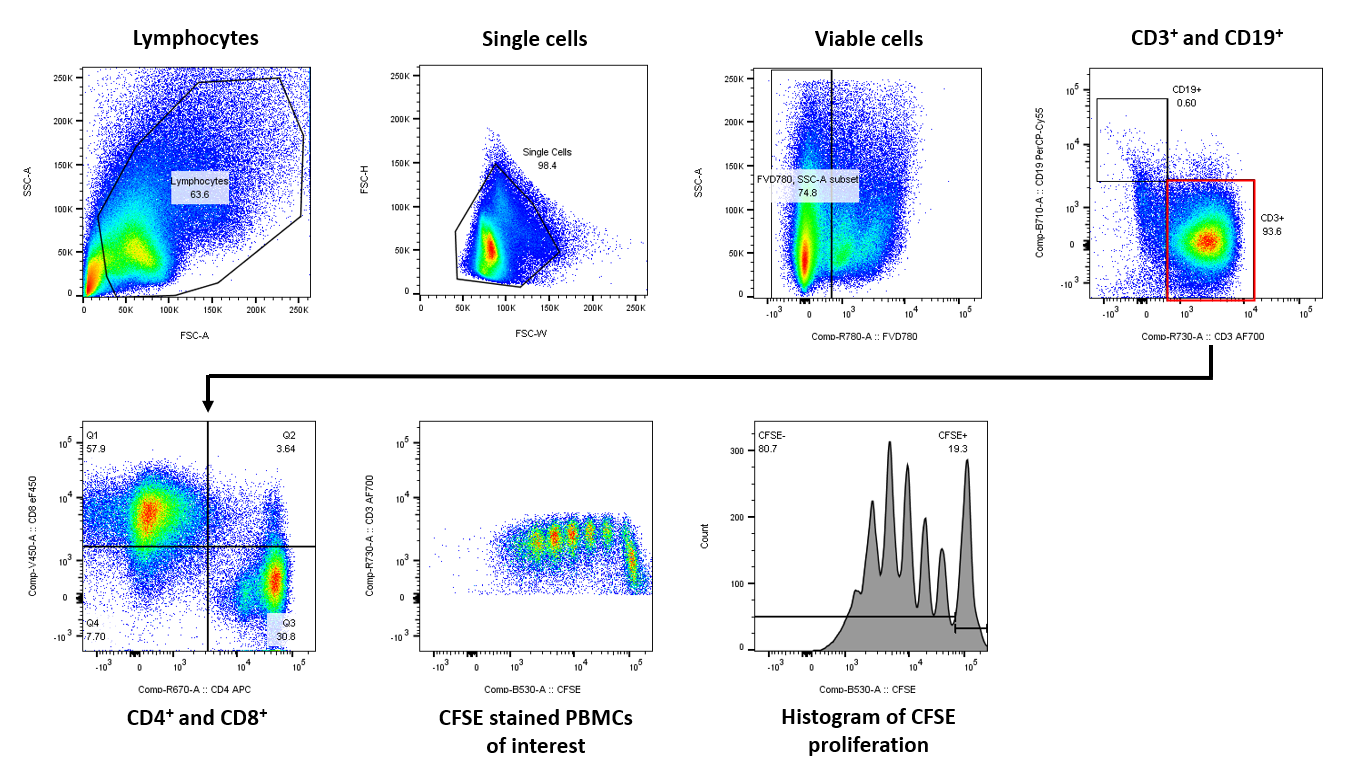

Supplement: Supplementary file 1 [file DataSheet_1.docx]
